# Remarks of Prof. McQuillen on Nitric Acid Treatment of Exposed Pulps

**Published:** 1859-10

**Authors:** J. Taft


### "REMARKS OF PROF. McQUILLEN ON NITRIC ACID TREATMENT OF EXPOSED PULPS."

BY J. T.

IN the September number of the *Cosmos*, we are somewhat sharply taken to account, because of the remarks we made in the August number of the "Register," upon the treatment of recently exposed pulps, by nitric acid. The *Cosmos* "feels impelled to protest emphatically against this kind of treatment." Well, the editor can *protest* as often as he chooses, but it is presumable that those who have tested and found this treatment successful, will continue to use it notwithstanding the protests. That the "vast majority of thorough operators in every part of the Union, had years ago abandoned as impracticable the effort to preserve the vitality of exposed pulps," is wholly new to us, and it is so new that we have not brought ourselves to the belief of it yet. It is true that the principal part of the profession in a few localities (one or two) have for the most part abandoned the treatment of exposed pulps with a view to their preservation. With them the simple exposure of a pulp is a loud call for its destruction. We have not so learned, from *precept* or *experience.* We have been treating exposed pulps, with a view to their preservation, by various methods for the last eighteen years, and the success has been such as to warrant a continuation. We did not abandon it because in some cases the desired end was not accomplished, but we endeavored to discriminate more closely, and treat more carefully.

The preservation of the vitality of the pulps of teeth is a desirable object, and one that should be sought even though an exposure of the pulp has been effected.

In the remarks we made upon the treatment of exposed pulps with nitric acid, we aimed simply to state a few plain, simple facts, in regard to the matter, and did not imagine that the statement could be made a matter of controversy. We stated the kind of treatment we employed in a specific class of cases, the method of accomplishing the work and the results.

Now, in regard to any of these, we have not much changed our opinion, we still continue this treatment, notwithstanding the castigation, and obtain results with which all concerned are well pleased. It is true that we have not employed this treatment a great while, but others in whom we have confidence, have been employing it for a number of years, and with very great success, and we have been led to experiment, and the results have been just as we stated. Within the last six months we have treated some twenty favorable cases, and all resulting well thus far; they are cases, the most of which we have seen since the operation, and any one of them giving trouble would be reported at once, for such cases were selected as we could control and such as would be reported.

There were several cases that we accounted unfavorable at the time of the operation, one-half or two-thirds of these were failures, but some of these are doing well as yet: They were unfavorable, either from a bad constitution, or some ill predisposition, long exposure, and irritation or inflammation of the pulp. Now if we have been employing this treatment, and we have these results, as we hope we are yet of sound mind sufficiently to determine, what will the "Cosmos" do about it?

Now we do object to having our head cut clean off at one swipe. We regret exceedingly that we are so far behind the times, as not to know that the "vast majority of thorough operators throughout the Union have abandoned as impracticable, &c." We always knew we were very ignorant, but then to have it told right out in the "Cosmos," knocked us all into a cocked-hat.

Well! we will never try to know any thing again! if we should somebody will know it before us, and more of it too.

What fools we have been these many years, blundering along, treating exposed pulps, when every body else had found it impracticable, and had abandoned it! We really begin to be afraid to do anything, lest the *vast* majority has found it impracticable, and abandoned it.

If it would not be impertinent, we would suggest that every part of the Union is not in Philadelphia.

The broom-corn straw is a good idea; we are thankful for it, and give due credit.

We shall report in future on the success of the nitric acid treatment,—and we shall endeavor to report correctly—notwithstanding the "*vast majority.*"

